# Supplementary material for: NEMO: cancer subtyping by integration of partial multi-omic data
Source: Bioinformatics. 2019 Jan 30;35(18):3348–56. doi: 10.1093/bioinformatics/btz058 (PMC6748715; doi:10.1093/bioinformatics/btz058)
Supplement: btz058_Supplementary_Data [file btz058_supplementary_data.zip › btz058-suppl_data/Supplementary File 1.docx]

Supplementary Materials for“NEMO: cancer subtyping by multi-omics integration”

Nimrod Rappoport and Ron Shamir^*^

The Blavatnik School of Computer Science, Tel Aviv University, Tel Aviv 69978, Israel

*To whom correspondence should be addressed. Tel: +972 3 640 5383; Fax: +972 3 640 5384;

Email: [rshamir@tau.ac.il](mailto:rshamir@tau.ac.il) (RS)

Contents

[Supplementary Figures 2](#_Toc524378854)

[Supplementary Figure 1 2](#_Toc524378855)

[Supplementary Figure 2 3](#_Toc524378856)

[Supplementary Figure 3 4](#_Toc524378857)

[Supplementary Figure 4 5](#_Toc524378858)

[Supplementary Figure 5 6](#_Toc524378859)

[Supplementary Figure 6 7](#_Toc524378860)

[Supplementary Figure 7 8](#_Toc524378861)

[Supplementary Figure 8 9](#_Toc524378862)

[Partial Data Simulation 10](#_Toc524378863)

[Hardware 11](#_Toc524378864)

[Datasets 12](#_Toc524378865)

[Benchmarked Methods and Software 13](#_Toc524378866)

[PVC 13](#_Toc524378867)

[MCCA 13](#_Toc524378868)

[Spectral Clustering 15](#_Toc524378869)

[Data Imputation 16](#_Toc524378870)

# Supplementary Tables

## Supplementary Table 1: Survival analysis results (-log10 of logrank’s p-value)

## Supplementary Table 2: Number of enriched clinical parameters (p ≤0.05)

## Supplementary Table 3: Number of clusters

## Supplementary Table 4: Runtime (seconds)

## Supplementary Table 5: Geometric mean runtime (seconds)

| kmeans | spectral | lracluster | pins | snf | mkl | mcca | nmf | iCluster | nemo |
| --- | --- | --- | --- | --- | --- | --- | --- | --- | --- |
| 281 | 4 | 2446 | 171 | 10 | 230 | 18 | 27 | 4024 | 9 |

## Supplementary Table 6: Results on the Handwritten dataset

|  | Full data | 50% missing from second omic |
| --- | --- | --- |
| NEMO | 0.73 (0) | 0.51 (0.03) |
| NEMO after imputation | 0.73 (0) | 0.37 (0.06) |
| rMKL-LPP | 0.77 (0.03) | 0.24 (0.02) |
| CCA with pixel representation | 0.51 (0.03) | 0.47 (0.02) |
| CCA with Fourier representation | 0.58 (0.01) | 0.08 (0.05) |
| PVC | 0.49 (0) | 0.28 (0.03) |

The Adjusted Rand Index of the clustering solution obtained by each of the algorithms and the true clustering of the Handwritten dataset. The results are the mean ARI across 10 repeats. The numbers in parentheses are one standard deviation.

# Supplementary Figures

## Supplementary Figure 1

Mean performance of the ten algorithms on ten cancer datasets. Blue bars are the measured average differential survival between clusters (-log10 logrank test's p-values). Red bars are the average number of enriched clinical parameters in the clusters. Bars indicate one standard deviation.

## Supplementary Figure 2

q
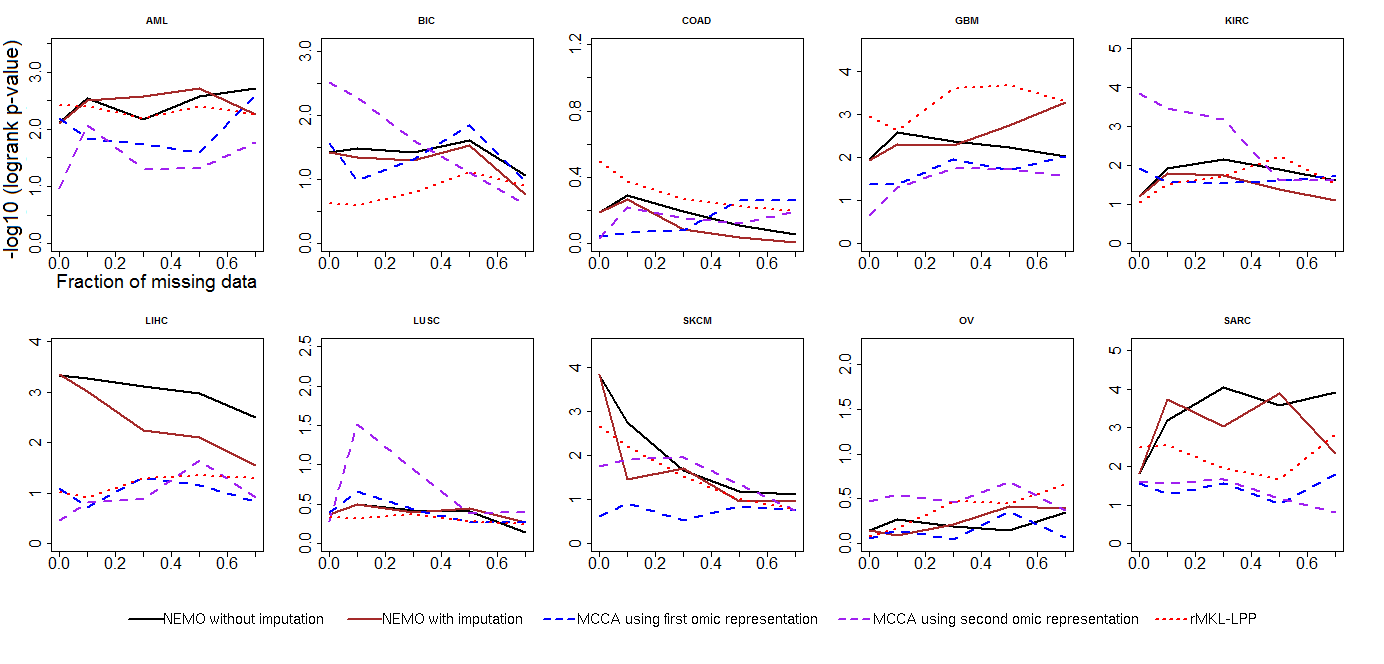


Performance on ten real cancer datasets with three omics (mRNA expression, DNA methylation and miRNA expression). The x-axis shows the fraction of patients for which gene expression data were removed. Y-axis is the –log10 p-value of the logrank test for differential survival.

## Supplementary Figure 3


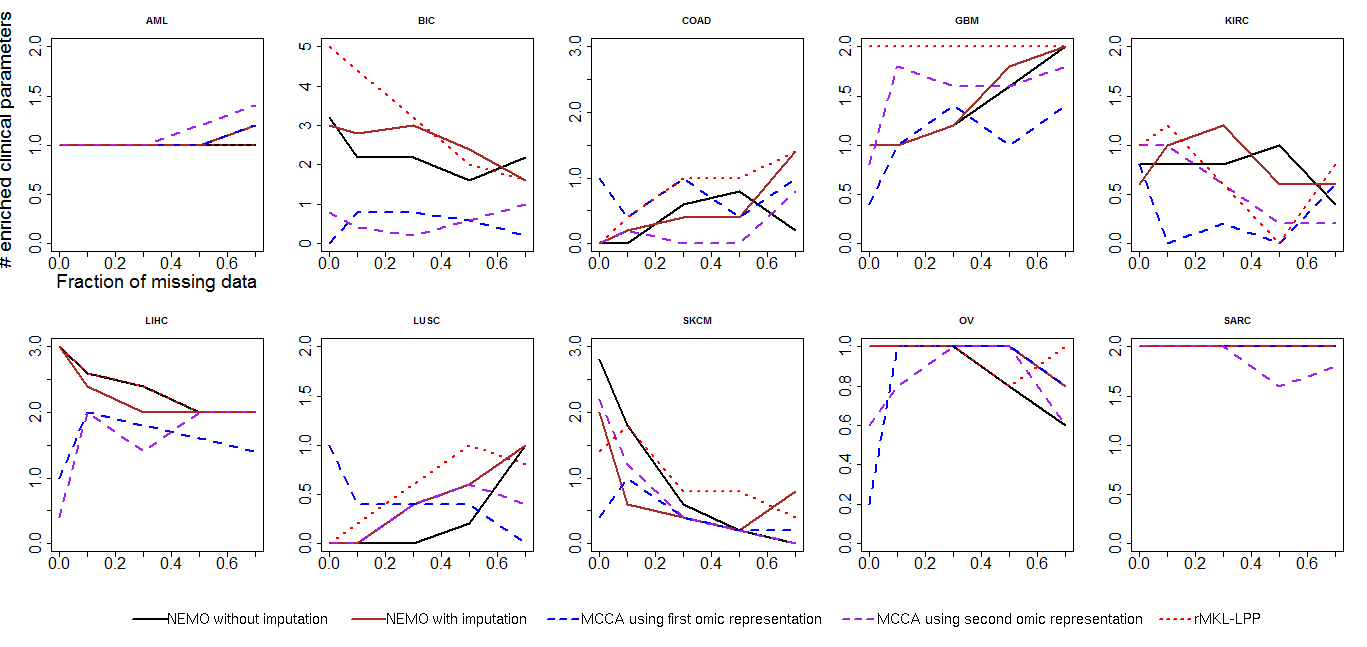


Performance on ten real cancer datasets with three omics (mRNA expression, DNA methylation and miRNA expression). The x-axis shows the fraction of patients for which gene expression data were removed. Y-axis is the number of enriched clinical parameters.

## Supplementary Figure 4


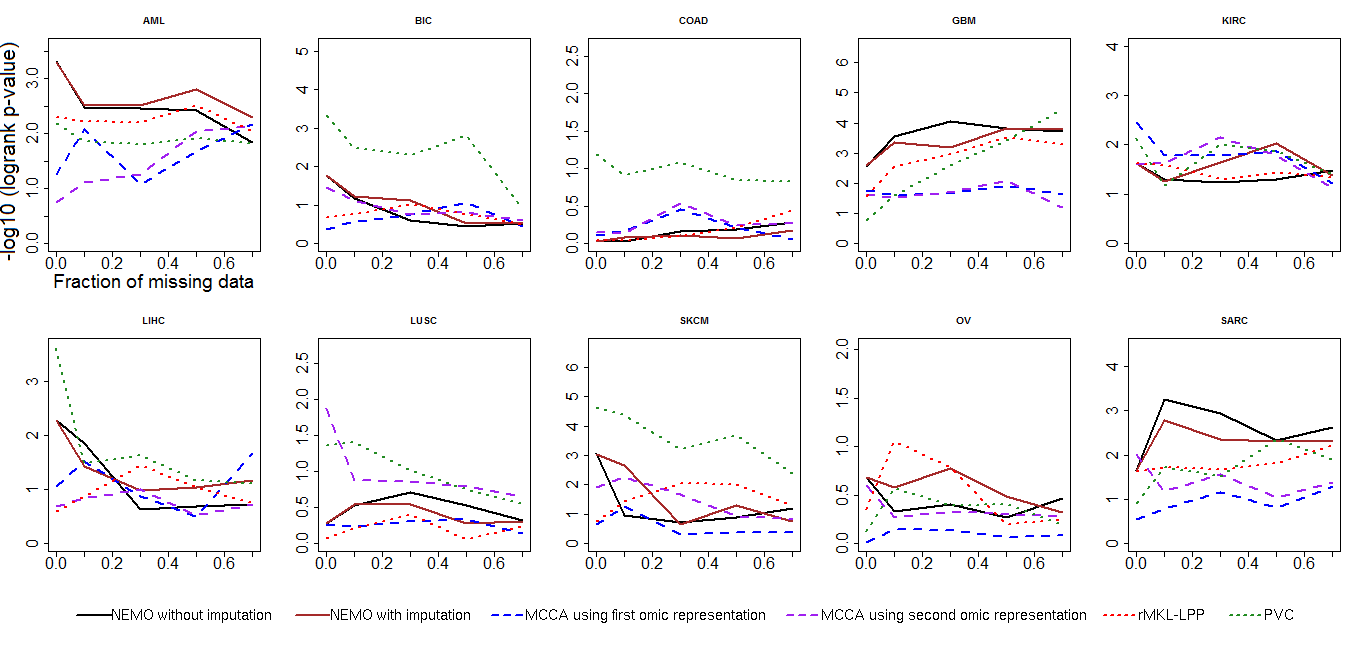


Performance on ten real cancer datasets with two omics (mRNA expression and DNA methylation). The x-axis shows the fraction of patients for which gene expression data were removed. Y-axis is the –log10 p-value of the logrank test for differential survival.

## Supplementary Figure 5


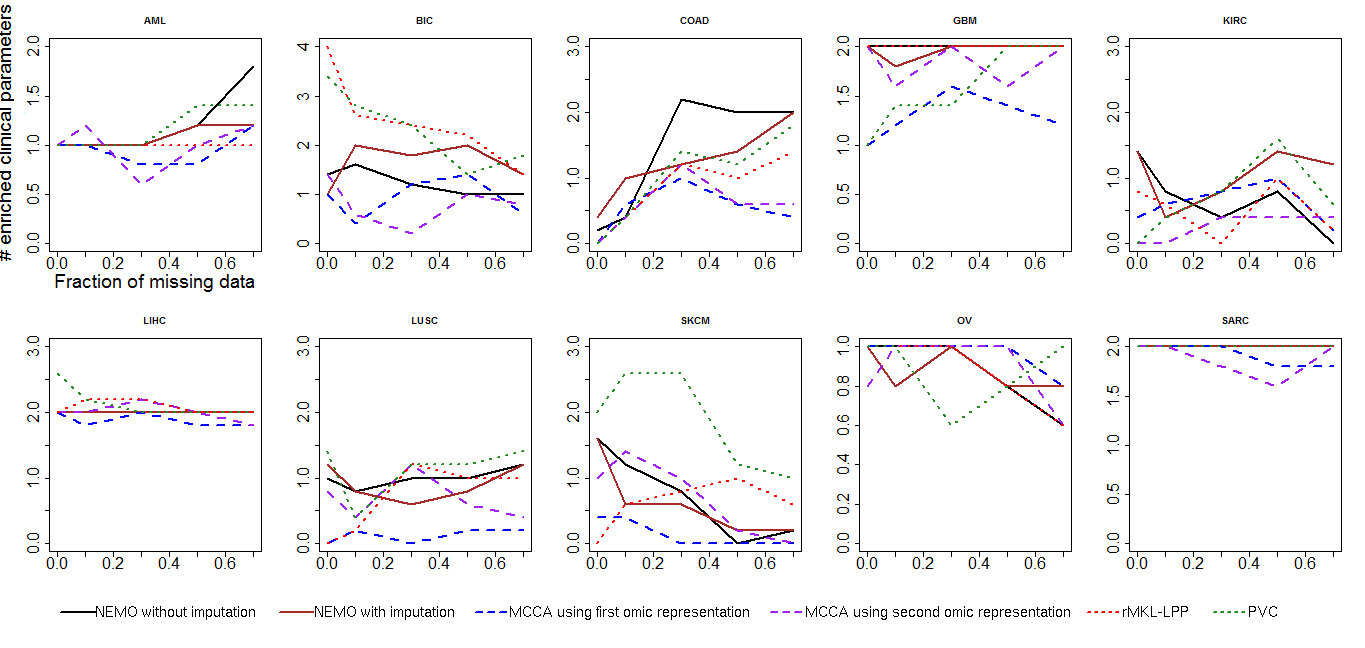


Performance on ten real cancer datasets with two omics (mRNA expression and DNA methylation). The x-axis shows the fraction of patients for which gene expression data were removed. Y-axis is the number of enriched clinical parameters.

## Supplementary Figure 6


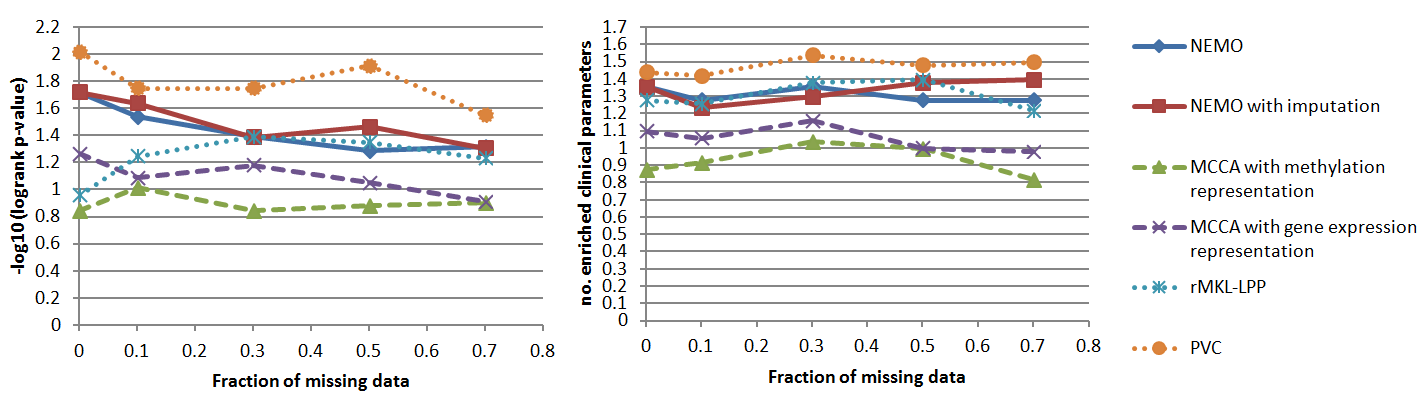


Performance with partial cancer data of two omics (mRNA expression and DNA methylation). The x-axis in both plots shows the fraction of patients for which gene expression data were removed. Y-axis in the left plot is the mean –log10 p-value of the logrank test. In the right plot it is the mean number of enriched clinical parameters. Results are average of ten real cancer datasets.

## Supplementary Figure 7


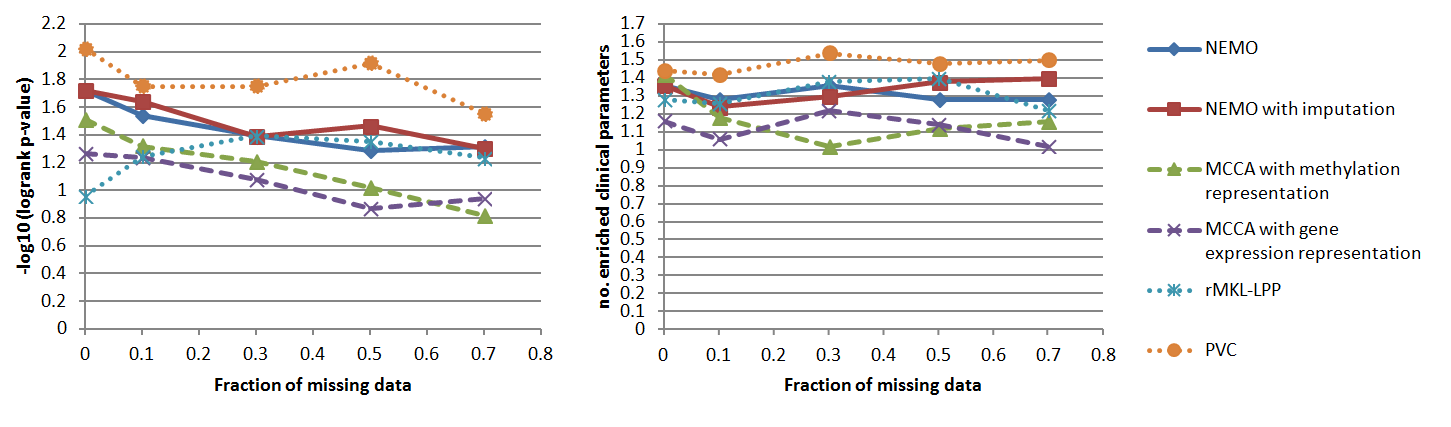


Performance with partial cancer data of two omics. This figure is the same as Supplementary Figure 6, but the order of omics in MCCA’s optimization is now (gene expression, methylation).

## Supplementary Figure 8


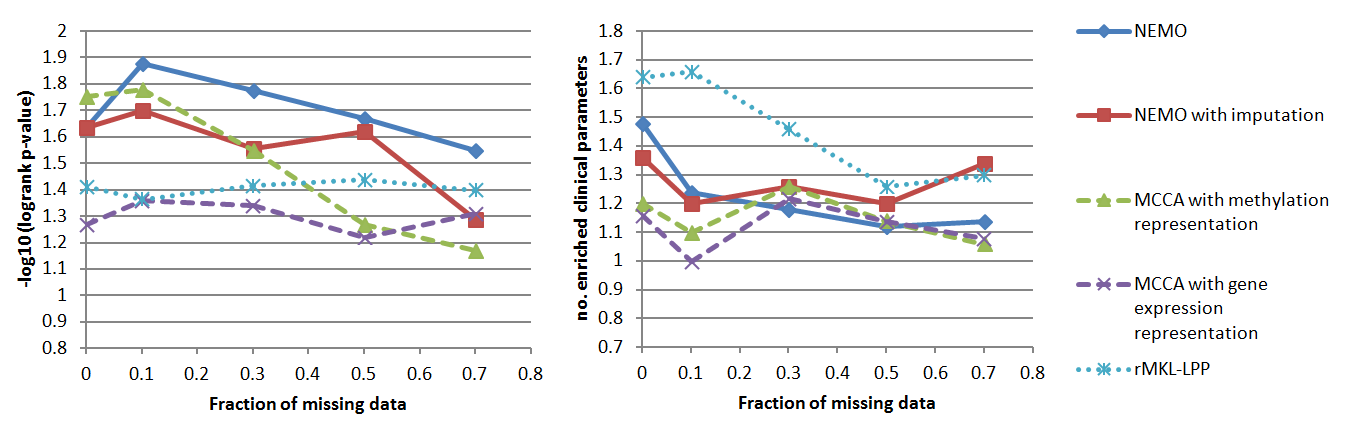


Performance on partial cancer datasets as a function of the fraction of samples missing data in one of the omics. This figure is the same as Figure 3, but the order of omics in MCCA’s optimization is now (gene expression, methylation, miRNA expression), as in the full datasets experiments.

## Supplementary Figure 9


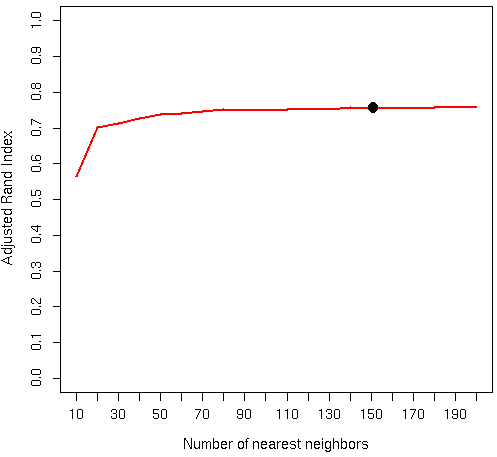


Robustness analysis for NEMO on simulated data. We ran NEMO using different values for *k*, the number of nearest neighbors used in the analysis, and report the Adjusted Rand Index compared to the correct clustering. The black dots indicate the point on the curve corresponding to the number of neighbors suggested by NEMO. The ARI reported here is the mean of 10 runs per each number of neighbors.

## Supplementary Figure 10


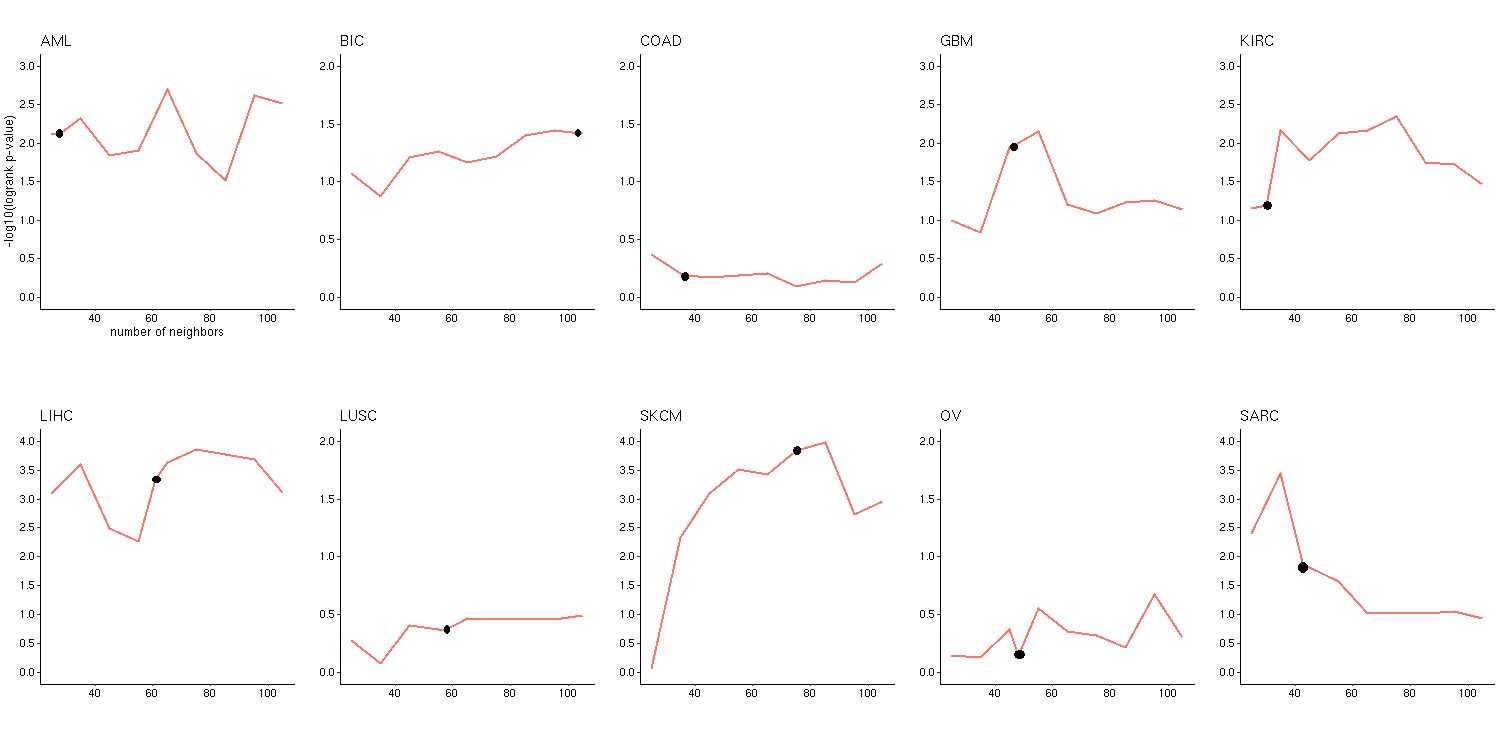


Robustness analysis for NEMO. For each TCGA dataset, we ran NEMO using different values for *k*, the number of nearest neighbors used in the analysis, and report the results of logrank test for comparing survival between the obtained clusters. Y-axis is -log10 of the logrank test p-value. Black dots indicate the point on the curve corresponding to the number of neighbors suggested by NEMO.

## Supplementary Figure 11


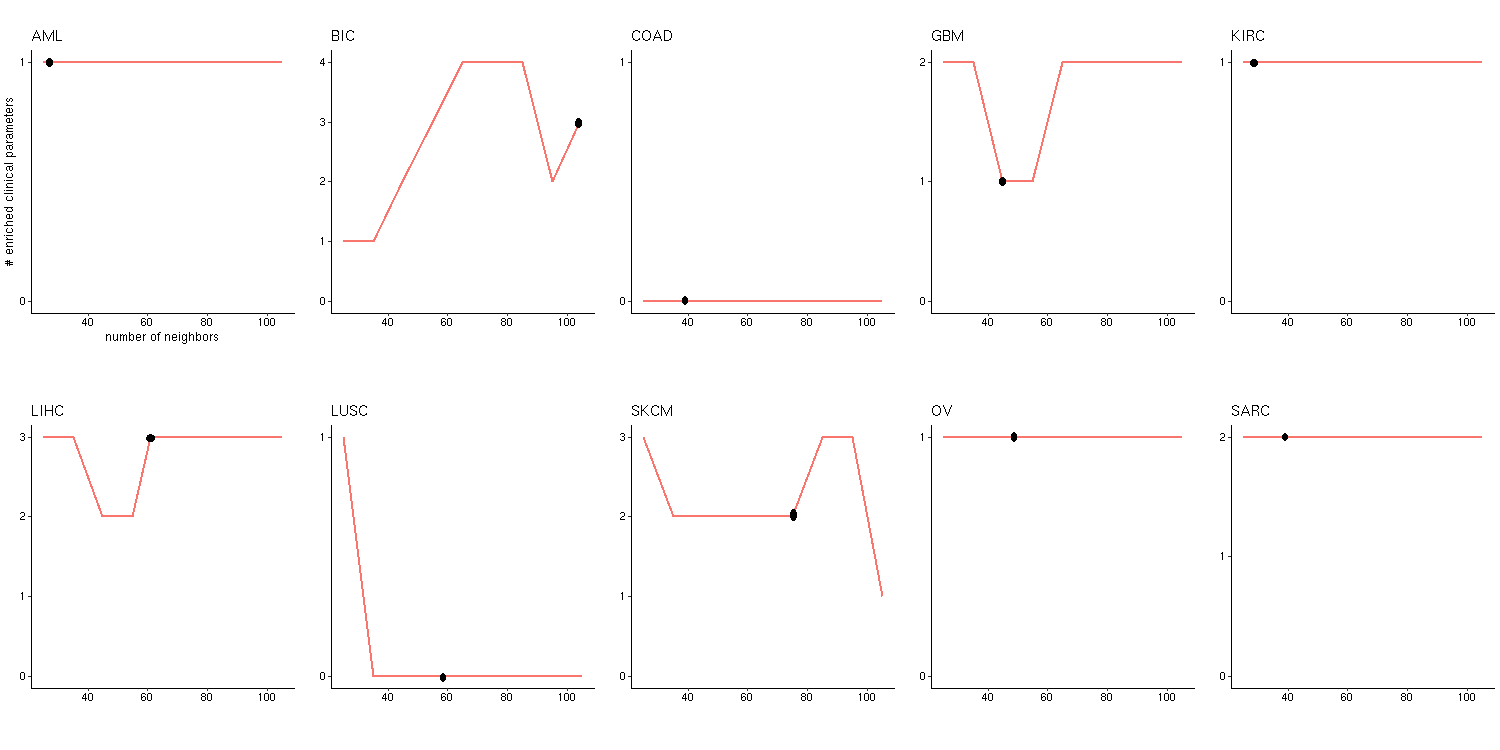


Robustness analysis for NEMO. For each TCGA dataset, we ran NEMO using different values for *k*, the number of nearest neighbors used in the analysis, and report the number of significantly enriched clinical parameters when comparing the obtained clusters. Black dots indicate the point on the curve corresponding to the number of neighbors suggested by NEMO.

## Supplementary Figure 12


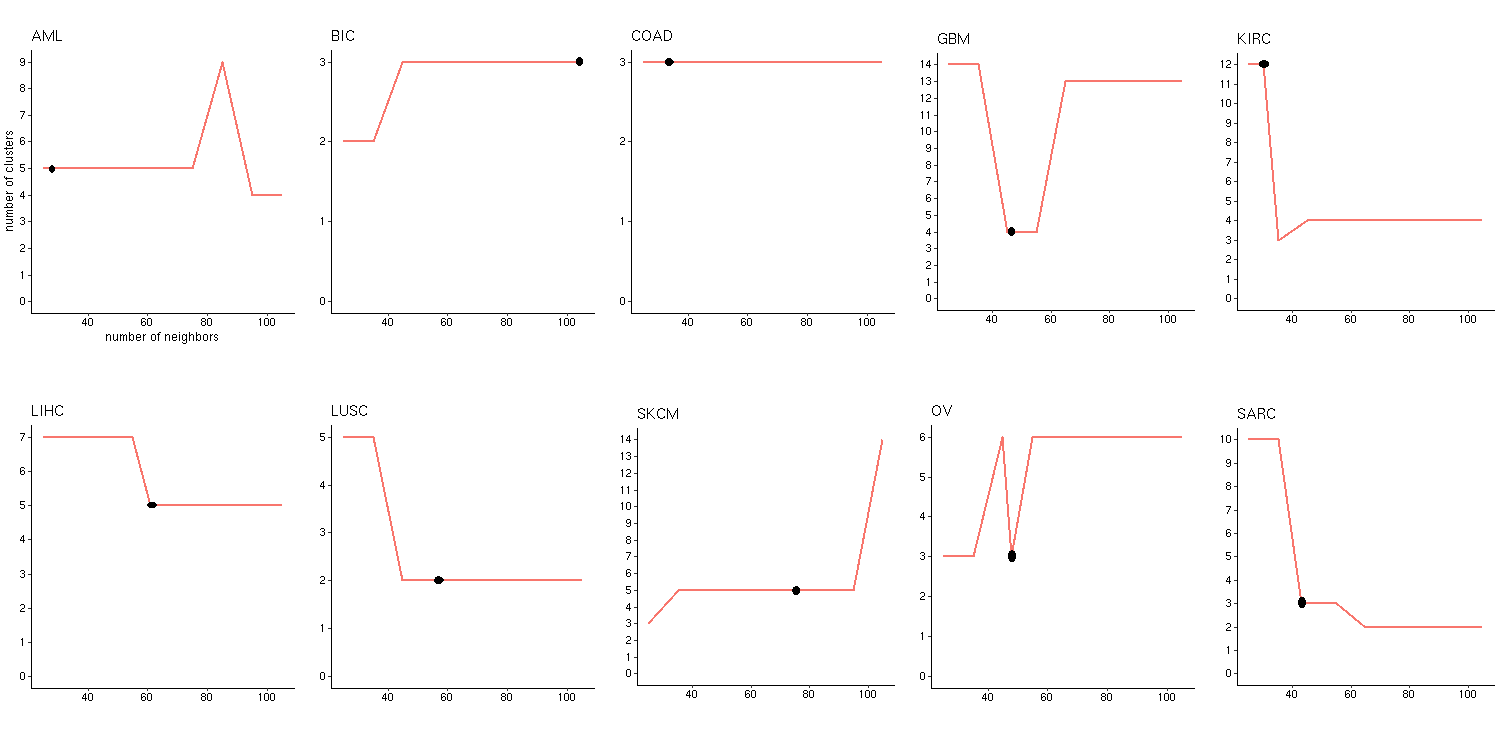


The number of clusters in NEMO as a function of the number of neighbors. For each TCGA dataset, we ran NEMO using different values for *k*, the number of nearest neighbors used in the analysis, and report the number of clusters determined by NEMO. Black dots indicate the point on the curve corresponding to the number of neighbors suggested by NEMO.

## Supplementary Figure 13


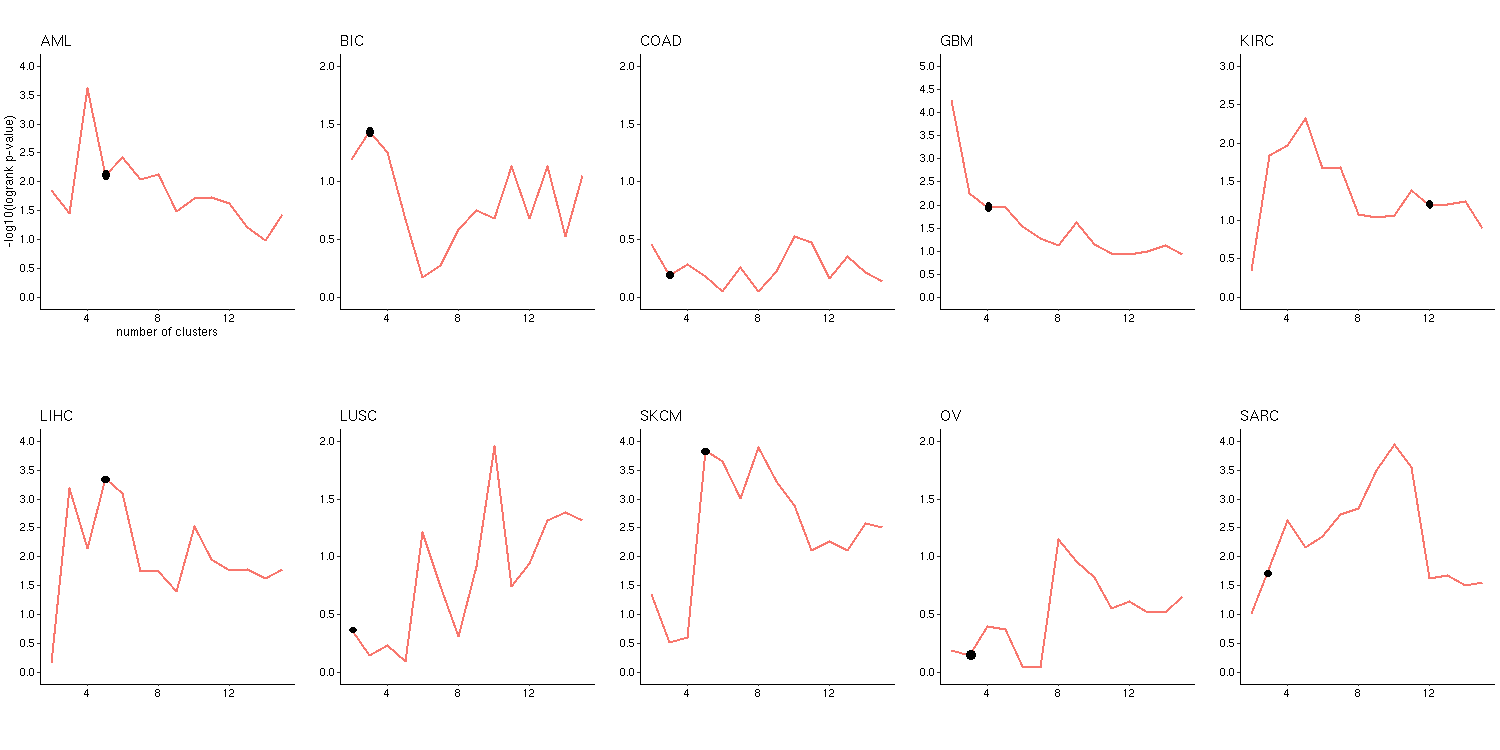


Robustness analysis for NEMO. For each TCGA dataset, we ran NEMO with a specified number of clusters, instead of choosing that number by the algorithm. We report the results of logrank test for comparing survival between the obtained clusters. Y-axis is -log10 of the logrank test p-value. Black dots indicate the point on the curve corresponding to the number of clusters suggested by NEMO using the eigengap method.

## Supplementary Figure 14


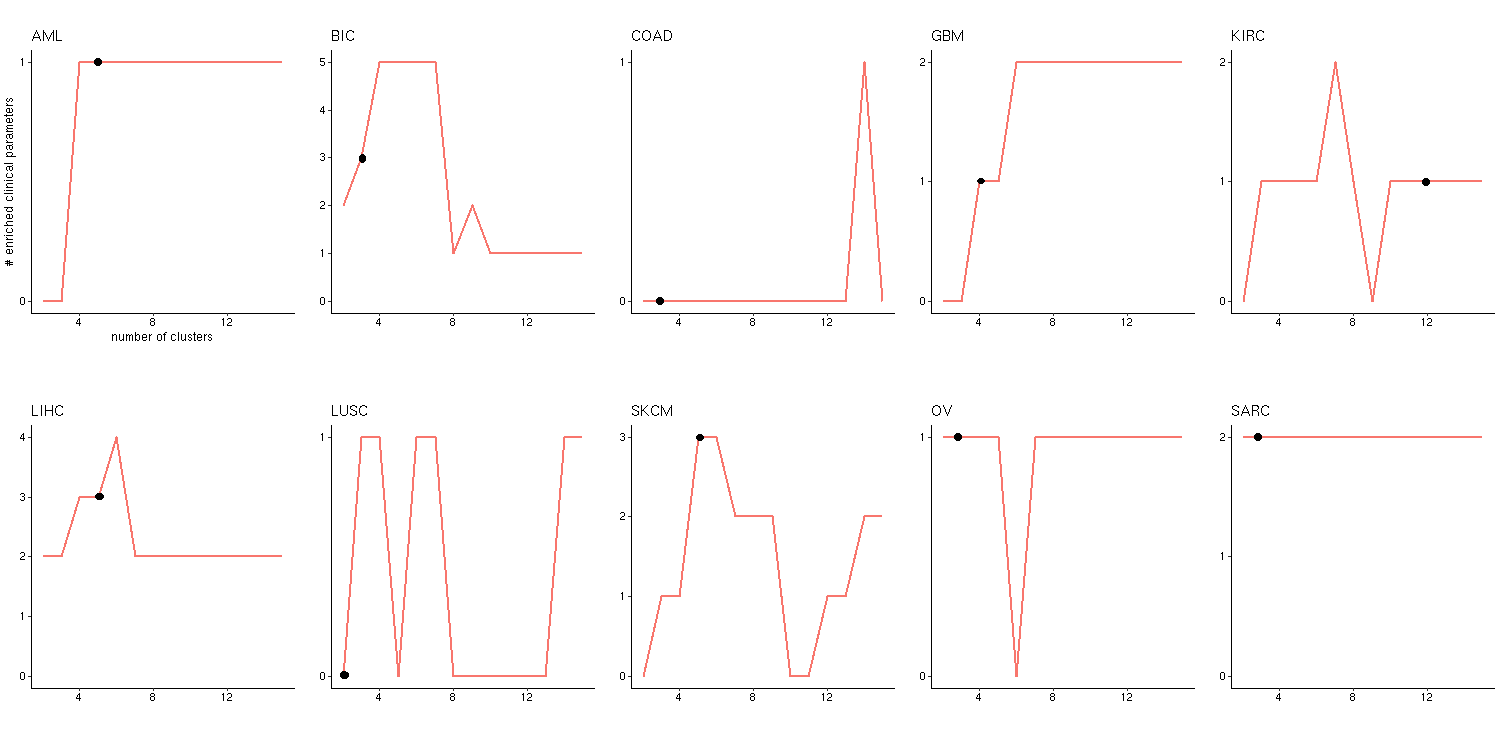


Robustness analysis for NEMO. For each TCGA dataset, we ran NEMO with a specified number of clusters, instead of choosing that number by the algorithm. We report the number of significantly enriched clinical parameters when comparing the obtained clusters. Black dots indicate the point on the curve corresponding to the number of clusters suggested by NEMO using the eigengap method.

# Partial Data Simulation

We simulated data in two scenarios, in order to gauge NEMO's performance in partial data settings, where the true underlying clustering is known. The simulations were designed to be simple and have a small number of parameters. In the first scenario there are two clusters and two omics. The first cluster has center $(0, ..., 0) \in R^{10}$ and the second $(0,0, 0, 0, 0, 2, 2, 2, 2, 2) \in R^{10}$. For each cluster, 150 sample vectors in $R^{10}$ are generated by sampling them around the center with multivariate Gaussian noise with mean zero and covariance matrix $I$, the identity matrix. We next create two omic profiles from this underlying cluster structure. Each omic profile is a vector in $R^{10}$ created by adding to the sample vector multivariate normal noise with mean zero and $2I$ covariance matrix. Partial data are created by removing the data of the second profile for a randomly selected subset of the samples.

One possible concern for NEMO is that averaging the similarities of different omics might cause omics with less noise to dominate the result. To test this, in the second scenario we simulated three omics. The first two omics are identical to the first simulation. The third is aimed to simulate an omic with no signal related to the cluster structure. This is done by sampling for both clusters normal vectors in $R^{10}$ with mean zero and $5I$ covariance matrix.

To compare NEMO to methods that do not support partial datasets, we imputed missing values by concatenating the different omics and using KNN imputation on the concatenated matrix.

We generated ten multi-omic datasets for each of the two scenarios. For each generated dataset, we randomly drew a fraction of the samples and removed their second omic data. The fraction varied from 0 to 0.8. For each fraction in each of the ten generated datasets we performed ten draws, so in total we tested 100 datasets per fraction in each scenario.

# Hardware

All experiments for timing the different methods were run on the following machine:

Windows 7 enterprise
Intel® Core™ i7-4770 CPU @ 3.4GHz
4 cores
32 GB RAM
64 bit operating system

The only exception were experiments for iClusterBayes, which were performed on a cluster:

Linux 4.9
72 CPUs, 2300 MHz each
756 GB RAM
64 bit operating system

# Datasets

The processed TCGA datasets and phenotype file were downloaded from:

<http://acgt.cs.tau.ac.il/multi_omic_benchmark/download.html>

Full details about the preprocessing are available in:

<https://www.biorxiv.org/content/early/2018/07/19/371120>

Metadata about the used datasets appears in Supplementary File 2.

The Handwritten dataset was downloaded from:

<https://archive.ics.uci.edu/ml/machine-learning-databases/mfeat/>

On 23 November 2018.

# Benchmarked Methods and Software

Clustering results for the datasets without missing data for all methods were downloaded from:

<https://www.biorxiv.org/content/early/2018/07/19/371120>

Full details about the execution of all methods appear there.

In addition to the methods described in the above link, we used PVC on partial data as follows:

PVC
Preprocessing:
Sequence features were log transformed. The 2000 features with highest variance from gene-expression and methylation omics were selected. In the miRNA omic, features with zero variance were filtered.
Execution details:
The method PVCclust from PVC package was used, with lambda=0.01 as recommended by the developers of PVC.
Parameter selection:
The number of clusters was set to be the same as the number determined by NEMO.
Implementation:
PVC was downloaded from Github on December 1st 2017 (commit 6177fc2ae940c9d1efa020309fe7a9dfe14c8c58) <https://github.com/nishantrai18/MultiViewNMF/tree/master/partialMV/PVC/code>

MCCA

MCCA was executed as described in:
<https://www.biorxiv.org/content/early/2018/07/19/371120>

With the following clarification:

MCCA is a joint dimension reduction method, where samples have several different low dimensional representations, one per omic. The authors state that any representation can be used for further analysis, but we observed a large difference in results based on the representation chosen. For the results on full datasets, we used the gene expression representation, since we have not yet observed the large difference in results. For the experiments on partial data, both for synthetic and cancer data, we applied MCCA twice, and each time took a representation of a different omic. We report results using both representations. For the synthetic data, the two omics were the two ones containing signal. For cancer data, we used DNA methylation and gene expression.

MCCA optimizes its objective function with respect to one omics at a time. The order of optimization therefore changes the final results, so we report here that order. For the full data results the order is: gene expression, DNA methylation, miRNA expression. For the experiments with cancer partial datasets, the order was (DNA methylation, gene expression) for two omics and (DNA methylation, gene expression, miRNA expression) for three. For synthetic partial data, the order is (first omic with clustering signal, second omic with signal from which data is removed, noisy omic).

# Spectral Clustering

To perform clustering on the integrated similarity graph, we used spectral clustering.

Given a symmetric similarity graph W, the graph normalized Laplacian is defined by ${L=D}^{-0.5}(D-W)D^{0.5}$, where D is a diagonal matrix where each diagonal entry is the sum of the corresponding row in W.

Normalized spectral clustering computes the first k eigenvectors of L, the normalized Laplacian, and arranges them in an n x k matrix U, where n is the number of samples, where the columns are the eigenvectors, and each row matches a single sample. Each sample is assigned to cluster c if in the row matching the sample in U, column c has the maximum value.

Normalized spectral clustering is a relaxation of Ncut problem, which searches for cuts on graph such that the cuts have low values, and the total weights within clusters are high.

For more information, see:

von Luxburg, U. (2007). A tutorial on spectral clustering. *Statistics and Computing*, 17(4), 395-416.

# Data Imputation

To compare NEMO to methods that do not support partial data, we first randomly sampled patients and removed the relevant omic data, and then imputed the missing data. The imputation was knn imputation on the concatenation of all single-omic matrices. We used the method knn.impute from the R package bnstruct with default parameters, except for settings cat.var=c() since we did not use categorical variables.
